# Morphology evolution and pure red upconversion mechanism of β-NaLuF_4_ crystals

**DOI:** 10.1038/srep28051

**Published:** 2016-06-16

**Authors:** Hao Lin, Dekang Xu, Anming Li, Dongdong Teng, Shenghong Yang, Yueli Zhang

**Affiliations:** 1 State Key Laboratory of Optoelectronic Materials and Technologies, School of Materials Science and Engineering, Sun Yat-Sen University, Guangzhou 510275, PR China

## Abstract

A series of β-NaLuF_4_ crystals were synthesized via a hydrothermal method. Hexagonal phase microdisks, microprisms, and microtubes were achieved by simply changing the amount of citric acid in the initial reaction solution. Pure red upconversion (UC) luminescence can be observed in β-NaLuF_4_:Yb^3+^, Tm^3+^, Er^3+^ and Li^+^ doped β-NaLuF_4_:20% Yb^3+^, 1% Tm^3+^, 20% Er^3+^. Based on the rate equations, we report the theoretical model about the pure red UC mechanism in Yb^3+^/Tm^3+^/Er^3+^ doped system. It is proposed that the pure red UC luminescence is mainly ascribed to the energy transfer UC from Tm^3+^:^3^*F*_4_ → ^3^*H*_6_ to Er^3+^:^4^*I*_11/2_ → ^4^*F*_9/2_ and the cross-relaxation (CR) effect [Er^3+^:^4^*S*_3/2_ + ^4^*I*_15/2_ → ^4^*I*_9/2_ + ^4^*I*_13/2_] rather than the long-accepted mechanism [CR process among Er^3+^:^4^*F*_7/2_ + ^4^*I*_11/2_ → ^4^*F*_9/2_ + ^4^*F*_9/2_]. In addition, compared to the Li^+^-free counterpart, the pure red UC luminescence in β-NaLuF_4_:20% Yb^3+^, 1% Tm^3+^, 20% Er^3+^ with 15 mol% Li^+^ doping is enhanced by 13.7 times. This study provides a general and effective approach to obtain intense pure red UC luminescence, which can be applied to other synthetic strategies.

Recently, lanthanide (Ln) doped upconversion (UC) materials have aroused extensive attention because of their potential applications in fields such as flat-panel displays, therapeutics, photovoltaics, and biological imaging^1^,^2^,^3^,^4^,^5^,^6^,^7^,^8^,^9^. Their advantages include long luminescence lifetimes, low toxicity and high photochemical stability^10^,^11^,^12^,^13^,^14^, which make them more desirable than conventional fluorescent materials. As an important fluoride, β-NaLuF_4_ has excellent UC luminescence due to its high refractive index, low phonon energy, and high thermal stability, which has attracted significant interest^15^,^16^,^17^,^18^. In contrast to green and blue light, red light (600–700 nm) can deeply penetrate biotissues owing to the lack of efficient endogenous absorbers^19^,^20^,^21^. Consequently, a strategy to achieve high purity of red UC luminescence will be useful for UC applications, especially for biological imaging. As is known, UC materials usually display multipeak emissions due to Ln ions have more than one metastable excited state^19^. Thus, avoiding the blue and green emissions and boosting the red emission are needed to obtain high pure red UC luminescence. For instance, Tan *et al*. reported the pure red UC emission in NaYbF_4_ nanocrystals doping with high Er^3+^ content^22^. Chan *et al*. revealed a UC mechanism in which energy transfer (ET) from Tm^3+^:^3^*F*_4_ → ^3^*H*_6_ to Er^3+^:^4^*I*_11/2_ → ^4^*F*_9/2_, leading to the population of red-emitting manifold (Er^3+^:^4^*F*_9/2_)^23^. Therefore, it can be concluded that high-content doping of Er^3+^ and low-content doping of Tm^3+^ can induce a tremendous increase in the red to green ratio (RGR) and ultimately pure red UC luminescence. There are three common cross-relaxation (CR) processes among Er^3+^ that account for the enhancement of RGR in Yb^3+^/Er^3+^ codoped system. The first CR effect is the long-accepted and most popular mechanism^22^,^24^,^25^,^26^. According to Capobianco *et al*.’s report^26^, the enhanced red UC emission was obtained owing to a CR process [Er^3+^:^4^*F*_7/2_ + ^4^*I*_11/2_ → ^4^*F*_9/2_ + ^4^*F*_9/2_], which directly populates the ^4^*F*_9/2_ state. The second CR effect was proposed by Gao *et al*.^27^, which was derived from Er^3+^:^4^*S*_3/2_ + ^4^*I*_13/2_ → ^4^*F*_9/2_ + ^4^*I*_11/2_, resulting in the promotion of red emission and quenching of green emission. The third CR effect was proposed by Salas *et al*.^28^, they reported that the increased red UC luminescence mainly comes from the CR process [Er^3+^:^4^*S*_3/2_ + ^4^*I*_15/2_ → ^4^*I*_9/2_ + ^4^*I*_13/2_], and subsequently energy transfer UC (ETU) process [*I*_13/2_ (Er^3+^) + ^2^*F*_5/2_ (Yb^3+^) → ^4^*F*_9/2_ (Er^3+^) + ^2^*F*_7/2_ (Yb^3+^)]. However, there is no theoretical model about the pure red UC mechanism in Yb^3+^/Tm^3+^/Er^3+^ doped system. Besides, compared to traditional phosphors, the main drawback of UC materials is their low UC luminescence efficiency. It is still a challenge to obtain the intense pure red UC luminescence, and an effective method to enhance the pure red UC emission is urgently needed. It has been proved that the doping of Li^+^ can greatly increase the UC luminescence intensity^29^,^30^,^31^. As is known, Li^+^ can be easily doped into the host lattice substitutionally or interstitially owing to its small ionic radius, which would reduce the symmetry of crystal field around Ln ions, inducing the enhancement of UC emission intensity. However, there is no report on the increase of pure red UC luminescence by introducing Li^+^ in β-NaLuF_4_:Yb^3+^, Tm^3+^, Er^3+^.

As a typical solution-based approach, the hydrothermal method has been widely applied to synthesize inorganic materials with controllable structures and morphologies^32^,^33^. During the hydrothermal treatment, a series of external parameters such as the pH value, citrate ions (Cit^3−^) content, NaF content, reaction time and temperature may have significant effects on the morphology evolution of particles^18^,^34^,^35^,^36^. In particular, the addition of chelating agent has a great impact on the kinetics of crystal growth^37^,^38^. Cit^3−^ as a shape modifier plays a critical role in the shape evolution of the final products due to its high thermal stability and ability to form complexes with other metal ions^39^,^40^,^41^.

In this article, a series of β-NaLuF_4_ crystals were prepared via a hydrothermal method using citric acid as a chelating agent, and their pure red UC luminescence were studied. Hexagonal phase microdisks, microprisms, and microtubes were achieved by simply changing the amount of citric acid in the initial reaction solution. Importantly, pure red UC luminescence can be observed in β-NaLuF_4_:Yb^3+^, Tm^3+^, Er^3+^ and Li^+^ doped β-NaLuF_4_:20% Yb^3+^, 1% Tm^3+^, 20% Er^3+^. Based on the rate equations, the theoretical model about the pure red UC mechanism in Yb^3+^/Tm^3+^/Er^3+^ doped system is presented. The red UC emission of 660 nm in Li^+^ doped β-NaLuF_4_:20% Yb^3+^, 1% Tm^3+^, 20% Er^3+^ is greatly increased compared to the Li^+^-free sample under 980 nm excitation at room temperature.

## Results and Discussion

### Morphology evolution of β-NaLuF_4_ crystals

**Figure 1:**
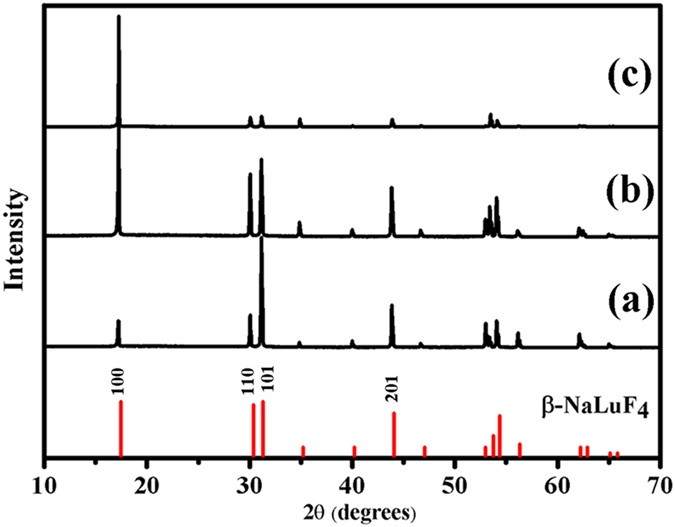
XRD patterns of β-NaLuF_4_ crystals with different citric acid contents. (**a**–**c**) refer to 2, 3, 8 mmol, respectively. The vertical red lines are the standard profiles of β-NaLuF_4_ (JCPDS 27-0726).

**Figure 2:**
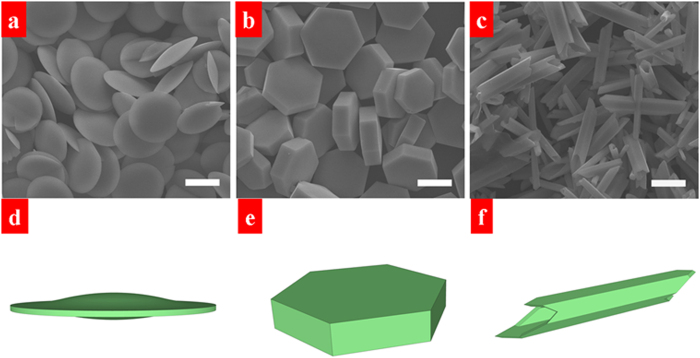
SEM images (**a**–**c**) and the corresponding schematic diagrams (**d**–**f**) of β-NaLuF_4_ crystals with different citric acid contents: (**a**,**d**) 2 mmol, (**b**,**e**) 3 mmol, and (**c**,**f**) 8 mmol. Scale bars = 5 μm.

Citric acid has been regarded as one of the most effective chelating agents because of its ability to regulate the morphology and dimension of the samples in the hydrothermal process^42^. In the present system, citric acid also plays a critical role in the morphology evolution of β-NaLuF_4_ crystals. Figure 1 shows the XRD patterns of the as-prepared β-NaLuF_4_ samples with different citric acid contents from 2 to 8 mmol. As can be seen, all the diffraction peaks can be well indexed to pure β-NaLuF_4_, which is consistent with the standard card (JCPDS 27-0726). No other impurity peaks are detected, indicating the high purity of β-NaLuF_4_ samples. It is worth to note that the relative intensities of (100), (110), (101) and (201) peaks display some differences from each other, implying the existence of oriented growth under different Cit^3−^ contents. The above XRD results are supported by the corresponding SEM images, as exhibited in Fig. 2. When the adding citric acid is 2 mmol (Fig. 2a), regular hexagonal phase microdisks with an average size of 0.79 μm in height and 7.58 μm in diameter are obtained. As the citric acid content increases to 3 mmol (Fig. 2b), short hexagonal phase microprisms with uniformity and smooth surfaces are achieved. The mean height and diameter of the prisms are 2.12 μm and 8.51 μm, respectively. Further increasing the citric acid content to 8 mmol, hexagonal phase microtubes with hollow structure are presented in Fig. 2c. The tubes have an average height of 9.47 μm and an average diameter of 1.88 μm. The ratios of height to diameter (*H*/*D* ratios) are calculated to be about 0.10, 0.25, and 5.04 when the adding citric acid is 2, 3, and 8 mmol. From the above analysis, it can be concluded that the *H*/*D* ratio is increased as the citric acid content increases from 2 to 8 mmol. Based on the high anisotropic structure of β-NaLuF_4_^43^, when the adding citric acid increases from 2 to 8 mmol, Cit^3−^ absorbs onto the {0001} facets more strongly than the 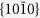 facets. Thus, the growth rate along [0001] direction is faster than that along 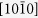 direction, resulting in the morphology evolution from disks to tubes and the enhancement of *H*/*D* ratio. The hollow structure of the tubes is generated owing to the growth rate at the center is lower than that at the edges^44^. The corresponding schematic diagrams of β-NaLuF_4_ crystals under different citric acid contents are displayed in Fig. 2(d–f).

### Pure red UC mechanism of β-NaLuF_4_:Yb^3+^, Tm^3+^, Er^3+^ crystals

**Figure 3:**
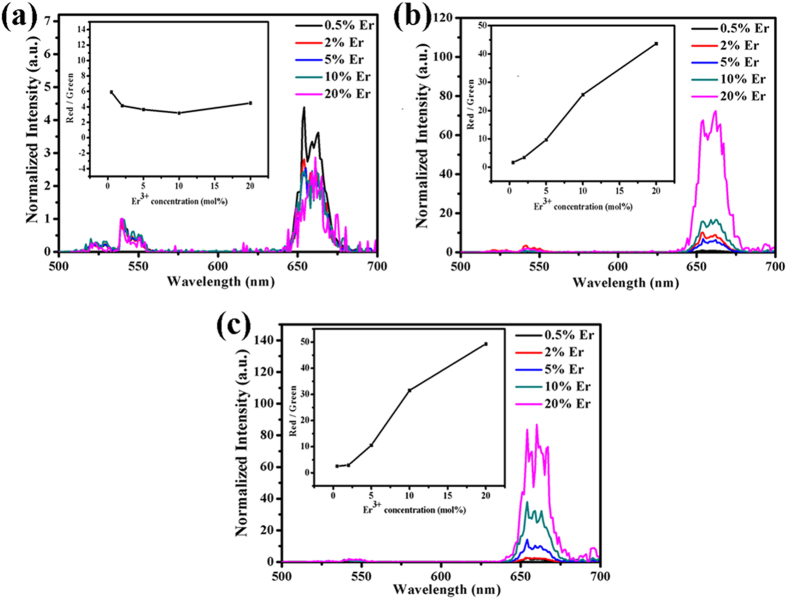
UC emission spectra (normalized to Er^3+^ 540 nm emission) of (**a**) β-NaLuF_4_:20% Yb/*x*Er, (**b**) β-NaLuF_4_:20% Yb/0.5% Tm/*x*Er, and (**c**) β-NaLuF_4_:20% Yb/1% Tm/*x*Er (*x* = 0.5, 2, 5, 10, 20%) under 980 nm excitation. The insets of (**a**–**c**) show the corresponding *R*/*G* ratio as a function of Er^3+^ concentration.

**Figure 4:**
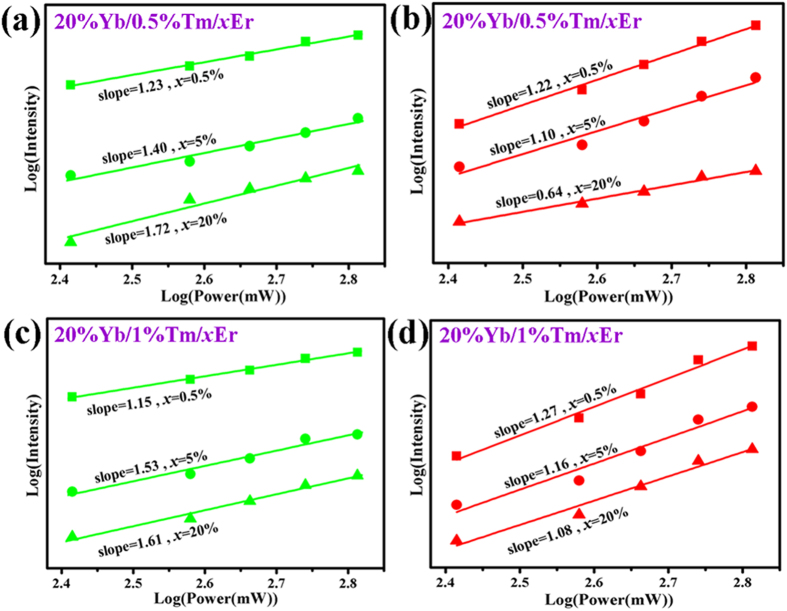
Double logarithmic relationship of (**a**,**c**) green and (**b**,**d**) red UC luminescence intensities versus pump powers in β-NaLuF_4_:20% Yb/0.5% Tm/*x*Er and β-NaLuF_4_:20% Yb/1% Tm/*x*Er (*x* = 0.5, 5, 20%) under 980 nm excitation.

A series of β-NaLuF_4_:Yb^3+^, Tm^3+^, Er^3+^ crystals were synthesized by adding 3 mmol citric acid. Figure 3 shows the UC emission spectra (normalized to Er^3+^ 540 nm emission) of (a) β-NaLuF_4_:20% Yb/*x*Er, (b) β-NaLuF_4_:20% Yb/0.5% Tm/*x*Er, and (c) β-NaLuF_4_:20% Yb/1% Tm/*x*Er (*x* = 0.5, 2, 5, 10, 20%) under 980 nm excitation at room temperature. Green emissions at around 520/540 nm correspond to the transitions of Er^3+^:^2^*H*_11/2_/^4^*S*_3/2_ → ^4^*I*_15/2_. Red emissions at approximately 660 nm and 696 nm are attributed to the transition of Er^3+^:^4^*F*_9/2_ → ^4^*I*_15/2_ and the transition of Tm^3+^:^3^*F*_3_ → ^3^*H*_6_, respectively. As can be seen, compared with Tm^3+^-free group [Fig. 3(a)], the RGR is greatly increased in 0.5% Tm^3+^-group [Fig. 3(b)] and 1% Tm^3+^-group [Fig. 3(c)]. The maximum RGR is observed in 0.5% Tm^3+^ and 1% Tm^3+^-groups doped with 20 mol% Er^3+^. From the insets of Fig. 3(a–c) and Table 1, it can be clearly seen that the RGR is almost unchanged in Tm^3+^-free group while dramatically enhanced in 0.5% Tm^3+^ and 1% Tm^3+^-groups with the increase of Er^3+^ dopant content. The RGR in 0.5% Tm^3+^ (R/G = 43.7) and 1% Tm^3+^ (R/G = 49.3)-groups with 20% Er^3+^ doping are increased by 26 and 19 times compared to their 0.5% Er^3+^ doping (R/G = 1.66, 2.57). Consequently, low-content doping of Tm^3+^ and high-content doping of Er^3+^ induce great enhancement in the RGR. Figure 4 shows the pump power dependence of green and red UC emissions in 0.5% Tm^3+^ and 1% Tm^3+^-groups with 0.5%, 5% and 20% Er^3+^ doping under 980 nm excitation. According to the formula^45^: *I*_uc_ ∝ *P*^n^, where *I*_uc_ is the output UC emission intensity, *P* is the infrared excitation power, n is the absorbed laser photon number when emitting an UC photon. As shown in Fig. 4(a,c), the slopes of green emission (Er^3+^:^2^*H*_11/2_/^4^*S*_3/2_ → ^4^*I*_15/2_) are 1.23, 1.40, and 1.72 in β-NaLuF_4_:20% Yb/0.5% Tm/*x*Er; 1.15, 1.53, and 1.61 in β-NaLuF_4_:20% Yb/1% Tm/*x*Er (*x* = 0.5, 5, 20%). The slopes of red emission (Er^3+^:^4^*F*_9/2_ → ^4^*I*_15/2_) are 1.22, 1.10, and 0.64 in 0.5% Tm^3+^-group; 1.27, 1.16, and 1.08 in 1% Tm^3+^-group with 0.5%, 5% and 20% Er^3+^ doping. On the basis of the above analysis, it can be concluded that green emission varies from one-photon process to two-photon processes, and red emission (660 nm) keeps one-photon process in 0.5% Tm^3+^ and 1% Tm^3+^-groups.

In this paper, we built a theoretical model to have a deep understanding of the ET process in Yb^3+^/Tm^3+^/Er^3+^ doped system. We supposed ^4^*F*_7/2_, ^2^*H*_11/2_, and ^4^*S*_3/2_ energy levels as a same level. When the Yb^3+^/Tm^3+^/Er^3+^ system doped with low Er^3+^ content, CR effect between Er^3+^ can be neglected. The corresponding rate equations are as follows:


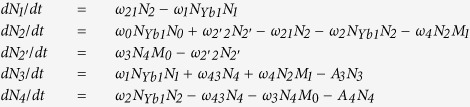


**Figure 5:**
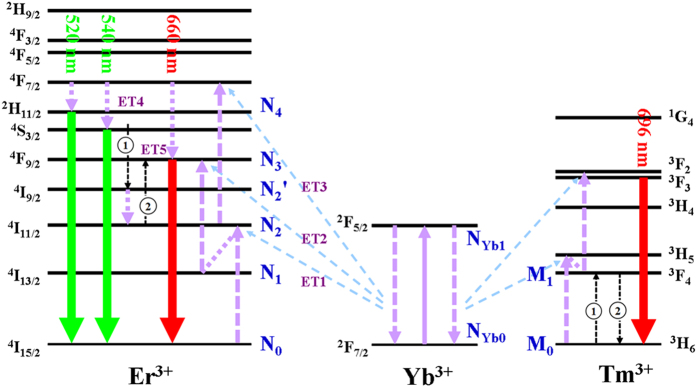
Proposed ET mechanism of green and red UC emissions in β-NaLuF_4_:Yb^3+^, Tm^3+^, Er^3+^ (with low Er^3+^ content). The mechanism involves the ETU from Tm^3+^ to Er^3+ ^23.

*N*_0_, *N*_1_, *N*_2_, *N*_2′_, *N*_3_, *N*_4_, *M*_0_, *M*_1_, *N*_Yb0_ and *N*_Yb1_ are the population densities of the Er^3+ 4^*I*_15/2_, ^4^*I*_13/2_, ^4^*I*_11/2_, ^4^*I*_9/2_, ^4^*F*_9/2_, ^4^*S*_3/2_/^2^*H*_11/2_/^4^*F*_7/2_, Tm^3+ 3^*H*_6_, ^3^*F*_4_, Yb^3+ 2^*F*_7/2_ and ^2^*F*_5/2_ levels, respectively. *ω*_0_, *ω*_1_, *ω*_2_, *ω*_3_, and *ω*_4_ correspond to the ET rates of ^2^*F*_5/2_ + ^4^*I*_15/2_ → ^2^*F*_7/2_ + ^4^*I*_11/2_ (ET1), ^2^*F*_5/2_ + ^4^*I*_13/2_ → ^2^*F*_7/2_ + ^4^*F*_9/2_ (ET2), ^2^*F*_5/2_ + ^4^*I*_11/2_ → ^2^*F*_7/2_ + ^4^*F*_7/2_ (ET3), ^4^*S*_3/2_ + ^3^*H*_6_ → ^4^*I*_9/2_ + ^3^*F*_4_ (ET4), and ^3^*F*_4_ + ^4^*I*_11/2_ → ^3^*H*_6_ + ^4^*F*_9/2_ (ET5), respectively. *ω*_21_, *ω*_2′2_, and *ω*_43_ are multiphonon relaxation (MPR) rates from Er^3+^:^4^*I*_11/2_ → ^4^*I*_13/2_, ^4^*I*_9/2_ → ^4^*I*_11/2_, and ^4^*F*_7/2_ → ^4^*F*_9/2_, respectively. *A*_3_ and *A*_4_ are the spontaneous radiative probabilities of Er^3+ 4^*F*_9/2_ and ^4^*S*_3/2_/^2^*H*_11/2_ levels, respectively. As presented in Fig. 5, the Er^3+ 4^*F*_7/2_ level is populated through the ET1 + ET3 processes. Then green UC emission is generated by the MPR processes of Er^3+^:^4^*F*_7/2_ → ^4^*S*_3/2_/^2^*H*_11/2_ levels. For red UC emission (660 nm), the Er^3+ 4^*F*_9/2_ level is populated in two ways: (a) the MPR from Er^3+ 4^*F*_7/2_ level, (b) the ET4+ET5 processes. In consideration of the high Yb^3+^ content, many radiative and nonradiative processes can be ignored, such as the back ET process from Er^3+^ to Yb^3+^ (^4^*I*_11/2_ + ^2^*F*_7/2_ → ^4^*I*_15/2_ + ^2^*F*_5/2_), MPR processes, *N*_1_, *N*_2_ radiative emissions and so on. Under steady-state condition, the rate equations can be acquired as follows:


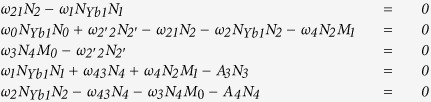


The population density of Yb^3+ 2^*F*_5/2_ can be described as follows^46^:


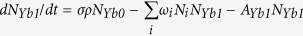


where *σ* is the absorption cross-section of Yb^3+ 2^*F*_5/2_ level, *ρ* is the pump rate of near-infrared (NIR) laser. Under steady-state condition, we get


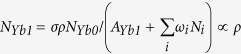


By solving the above equations, we have


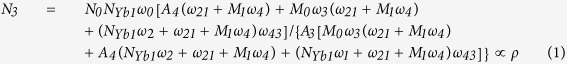



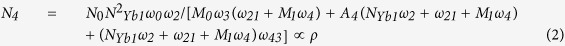



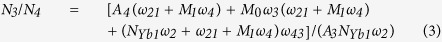


As can be seen from Equations (1) and (2), both red-emitting manifold *N*_3_ and green-emitting manifold *N*_4_ have linear relationships with pump power at low Er^3+^ dose, which are in good agreement with the experimental results shown in Fig. 4.

**Figure 6:**
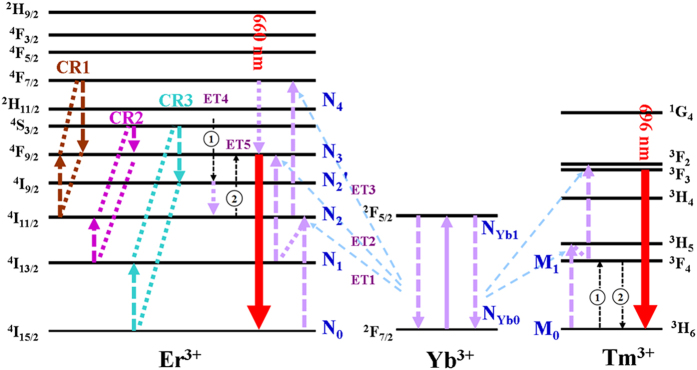
Proposed ET mechanism of red UC emission in β-NaLuF_4_:Yb^3+^, Tm^3+^, Er^3+^ (with high Er^3+^ content). The mechanism involves the CR effects among Er^3+ ^26,27,28,46 and ETU from Tm^3+^ to Er^3+ ^23.

When the Yb^3+^/Tm^3+^/Er^3+^ system doped with high Er^3+^ dose, pure red UC luminescence can be obtained. As is known, CR is dependent on the distance among activators^46^. The average distance between Er^3+^ reduces with the increase of Er^3+^ dopant dose, which would result in the enhancement of CR effect. Thus, the CR process among Er^3+^ plays an important role in the achievement of pure red UC luminescence. There are generally three CR processes between Er^3+^ that account for the increase of RGR (has been described in the section of “Introduction”), as displayed in Fig. 6. Additionally, the ET4+ET5 processes between Tm^3+^ and Er^3+^ also make a significant contribution to the high RGR. In the following sections, the above three CR effects are discussed systematically. As the Yb^3+^/Tm^3+^/Er^3+^ system doped with high Er^3+^ content, green UC emission can be neglected, and red UC emission (660 nm) mainly comes from two ways: (a) the CR effect among Er^3+^; (b) the ETU from Tm^3+^ to Er^3+^.

### Theoretical model for the first CR effect

The corresponding rate equations are as follows:


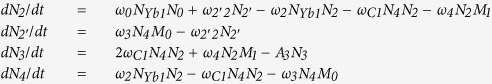


*ω*_C1_ is the CR rate for [Er^3+^:^4^*F*_7/2_ + ^4^*I*_11/2_ → ^4^*F*_9/2_ + ^4^*F*_9/2_ (CR1)]. CR1 is the long-accepted and most popular mechanism to account for the enhancement of RGR in Yb^3+^/Er^3+^ codoped system^22^,^24^,^25^,^26^. By solving the equations under steady-state excitation, we get


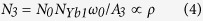



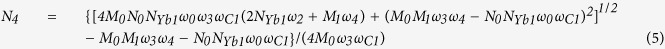



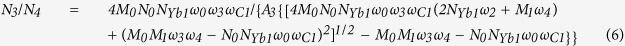


As can be seen from Equation (5), the green-emitting level *N*_4_ does not have quasi-quadratic relationship with pump power, which is not corresponding to Fig. 4(a,c) where n are 1.72 and 1.61 in 0.5% Tm^3+^ and 1% Tm^3+^-groups at high content (20 mol%) of Er^3+^ doping, indicating CR1 is not suitable for explaining the experimental results. Consequently, CR1 has nothing to do with the increase of red emission (660 nm) at high Er^3+^ content in Yb^3+^/Tm^3+^/Er^3+^ doped system.

### Theoretical model for the second CR effect

The corresponding rate equations are as follows:


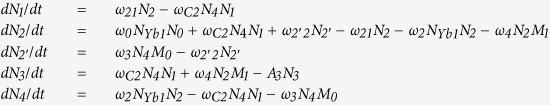


*ω*_C2_ is the CR rate for [Er^3+^:^4^*S*_3/2_ + ^4^*I*_13/2_ → ^4^*F*_9/2_ + ^4^*I*_11/2_ (CR2)]. By solving the equations under steady-state condition, we have


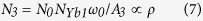



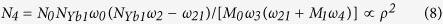



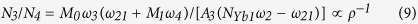


**Figure 7:**
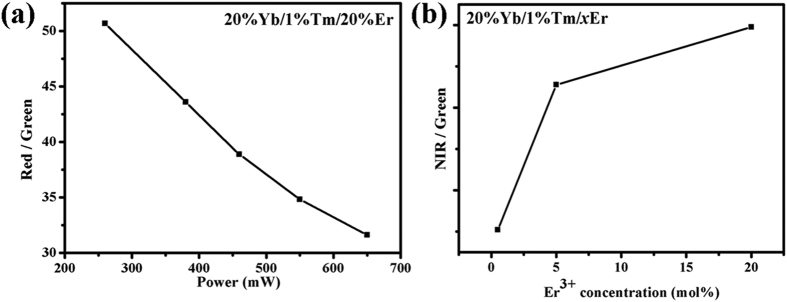
(**a**) Power dependence of *R*/*G* ratio for β-NaLuF_4_:20% Yb/1% Tm/20% Er, and (**b**) Er^3+^ concentration dependence of *NIR*/*G* ratio for β-NaLuF_4_:20% Yb/1% Tm/*x*Er under 980 nm excitation. NIR emission corresponds to the ^4^*I*_13/2_ → ^4^*I*_15/2_ transition of Er^3+^.

As can be seen from Equations (7) and (8), red-emitting manifold *N*_3_ and green-emitting manifold *N*_4_ have linear and quasi-quadratic relationships with pump power at high Er^3+^ content, which correspond to the relevant results in Fig. 4. The power dependence of RGR for β-NaLuF_4_:20% Yb/1% Tm/20% Er is exhibited in Fig. 7(a). It can be clearly seen that the RGR is decreased with the increase of pump power. The ratio of *N*_3_ to *N*_4_ shows the inverse proportional relationship to pump power [Equation (9)], which is in accordance with the result in Fig. 7(a). From the above analysis, it can be deduced that CR2 maybe makes a contribution to the high RGR when the Yb^3+^/Tm^3+^/Er^3+^ system doped with high Er^3+^ dose.

### Theoretical model for the third CR effect

The corresponding rate equations are as follows:


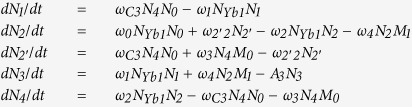


*ω*_C3_ is the CR rate for [Er^3+^:^4^*S*_3/2_ + ^4^*I*_15/2_ → ^4^*I*_9/2_ + ^4^*I*_13/2_ (CR3)], and red UC emission (660 nm) is obtained by the subsequent ETU process [*I*_13/2_ (Er^3+^) + ^2^*F*_5/2_ (Yb^3+^) → ^4^*F*_9/2_ (Er^3+^) + ^2^*F*_7/2_ (Yb^3+^)]. By solving the equations under steady-state condition, we get


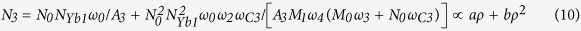



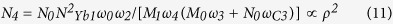



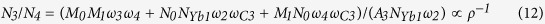


As can be seen from Equation (10), red-emitting level *N*_3_ ~ *aρ* + *bρ*^2^, which is corresponding to the relevant results in Fig. 4(b,d) when we suppose the parameter “b” is close to zero. Equations (11) and (12) show the green-emitting manifold *N*_4_ and ratio of *N*_3_ to *N*_4_ have quasi-quadratic and inverse proportional relationships with pump power, which are in good agreement with the results shown in Figs 4(a,c) and 7(a), respectively. Thus, CR3 can be used to explain the experimental results.

**Figure 8:**
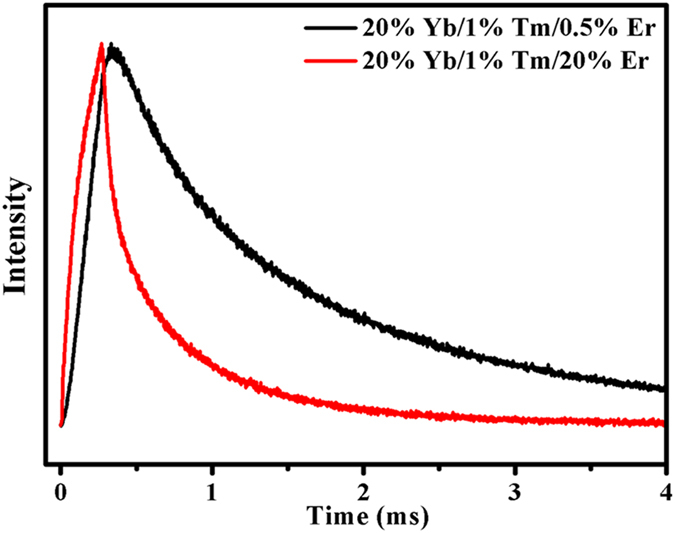
Decay curves of the ^4^*I*_13/2_ → ^4^*I*_15/2_ transition of Er^3+^ in β-NaLuF_4_:20% Yb/1% Tm/0.5% Er and β-NaLuF_4_:20% Yb/1% Tm/20% Er.

On the basis of the above analysis, it can be concluded that both CR2 and CR3 maybe are the appropriate ET mechanisms for the achievement of pure red UC luminescence at high Er^3+^ content in Yb^3+^/Tm^3+^/Er^3+^ doped system. According to our experimental results, there are three reasons to prove that CR3 (Er^3+^:^4^*S*_3/2_ + ^4^*I*_15/2_ → ^4^*I*_9/2_ + ^4^*I*_13/2_) is the main CR effect for the population process of ^4^*F*_9/2_ manifold. First, the ratio of NIR to green (NGR) is enhanced with the increase of Er^3+^ content in β-NaLuF_4_:20% Yb/1% Tm/*x*Er (NIR emission corresponds to the ^4^*I*_13/2_ → ^4^*I*_15/2_ transition of Er^3+^), as presented in Fig. 7(b). The increasing NGR indicates that the population of Er^3+ 4^*I*_13/2_ level becomes larger and larger compared to Er^3+ 4^*S*_3/2_/^2^*H*_11/2_ levels. As is known, the probability of MPR from ^4^*I*_11/2_ to ^4^*I*_13/2_ is quite low due to the low phonon energy in our system. Thus, the increasing NGR is mainly ascribed to CR3. Second, the decay curves of the ^4^*I*_13/2_ → ^4^*I*_15/2_ transition of Er^3+^ in β-NaLuF_4_:20% Yb/1% Tm/0.5% Er and β-NaLuF_4_:20% Yb/1% Tm/20% Er are shown in Fig. 8. The decay lifetime was calculated based on the function: 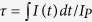, where *I*(t) is the emission intensity at time t, and *I*_P_ is the peak intensity in the decay curve. The calculation results show that τ_0.5% Er_ = 1.40 ms and τ_20% Er_ = 0.55 ms. The energy transfer efficiency (ETE) can be evaluated by the following expression: *η*_*ETE*_ = *1* − *τ*_*20*% Er_/*τ*_*0.5*% Er_, the calculation result shows that *η*_ETE_ = 60.71%. With the increase of Er^3+^ dose, the enhanced red UC emission (660 nm) mainly comes from the enhancement of CR effect between Er^3+^ in Yb^3+^/Tm^3+^/Er^3+^ doped system. Thus, it is reasonable to consider that the probability of CR3 is 60.71%. Third, the ET process of CR2 (Er^3+^:^4^*S*_3/2_ + ^4^*I*_13/2_ → ^4^*F*_9/2_ + ^4^*I*_11/2_) needs the population process of Er^3+ 4^*I*_13/2_ manifold, which is mainly dependent on the ET process of CR3 (Er^3+^:^4^*S*_3/2_ + ^4^*I*_15/2_ → ^4^*I*_9/2_ + ^4^*I*_13/2_). Therefore, the CR3 process is required before the CR2 process, resulting in the leading role of CR3 in the CR effects.

It can be concluded that the pure red UC luminescence is mainly ascribed to the ETU from Tm^3+^:^3^*F*_4_ → ^3^*H*_6_ to Er^3+^:^4^*I*_11/2_ → ^4^*F*_9/2_ and the CR effect [Er^3+^:^4^*S*_3/2_ + ^4^*I*_15/2_ → ^4^*I*_9/2_ + ^4^*I*_13/2_ (CR3)] rather than the long-accepted and most popular mechanism (CR1 process among Er^3+^:^4^*F*_7/2_ + ^4^*I*_11/2_ → ^4^*F*_9/2_ + ^4^*F*_9/2_).

### Li^+^ doped β-NaLuF_4_:20% Yb^3+^, 1% Tm^3+^, 20% Er^3+^ crystals

**Figure 9:**
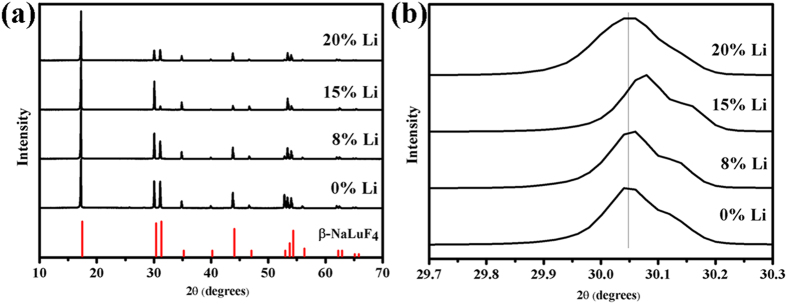
XRD patterns (**a**) and the main diffraction peak (**b**) of different Li^+^ doped β-NaLuF_4_:20% Yb^3+^, 1% Tm^3+^, 20% Er^3+^ crystals. The vertical red lines are the standard profiles of β-NaLuF_4_ (JCPDS 27-0726).

**Figure 10:**
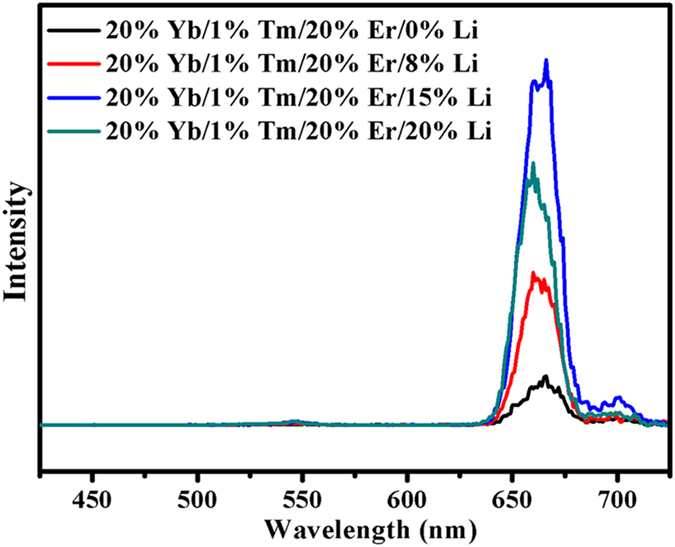
UC emission spectra of β-NaLuF_4_:20% Yb^3+^, 1% Tm^3+^, 20% Er^3+^ crystals doped with different Li^+^ contents under 980 nm excitation.

**Figure 11:**
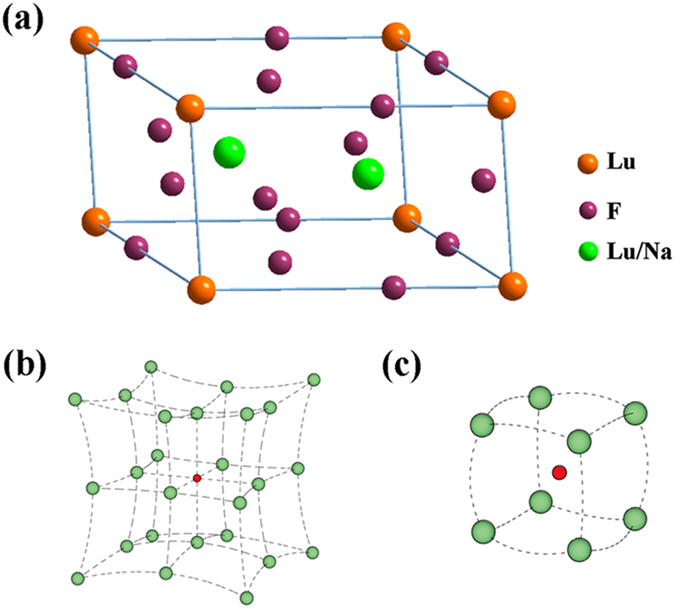
Crystal structure of β-NaLuF_4_ (**a**); possible changes in β-NaLuF_4_ crystal lattice after Li^+^ doping: substitution by Li^+^ (**b**), and interstitial occupation by Li^+^ (**c**).

A series of Li^+^ doped β-NaLuF_4_:20% Yb^3+^, 1% Tm^3+^, 20% Er^3+^ crystals were synthesized by adding 3 mmol citric acid. Figure 9 presents the XRD patterns (a) and the main diffraction peak (b) of different Li^+^ doped β-NaLuF_4_:20% Yb^3+^, 1% Tm^3+^, 20% Er^3+^ crystals. As shown in Fig. 9(a), all the diffraction peaks of the products can be indexed as pure β-NaLuF_4_ (JCPDS 27-0726) even the Li^+^ concentration increases up to 20 mol%, indicating that Li^+^ doping has no influence on the crystal structure of the products. The corresponding UC emission spectra of the products under 980 nm excitation are shown in Fig. 10. As can be seen, the pure red UC luminescence is greatly enhanced after Li^+^ doping. Compared to the Li^+^-free sample, the pure red UC luminescence in β-NaLuF_4_:20% Yb^3+^, 1% Tm^3+^, 20% Er^3+^ with 15 mol% Li^+^ doping is increased by 13.7 times. This phenomenon is mainly caused by the asymmetric surrounding environment around Ln ions after Li^+^ doping. Figure 9(b) exhibits that the main diffraction peak moves to the larger angles when the Li^+^ concentration is from 0 to 15 mol%, whereas shifts in reverse as the Li^+^ concentration increases up to 20 mol%. According to Bragg’s law 2*d*sin*θ* = *nλ*, where *d* represents the interplanar distance, *θ* represents the diffraction angle, and *λ* represents the diffraction wavelength. When *d* decreases, *θ* increases; when *d* increases, *θ* decreases. As is displayed in Fig. 11(a), Na^+^ and Ln^3+^ occupy the same lattice site in β-NaLuF_4_ lattice. When the Li^+^ is introduced into the host lattice, it can replace Na^+^ (*d* decreases, *θ* increases, 0 < Li^+^ concentration ≤15 mol%) [Fig. 11(b)] or occupy the interstitial site (*d* increases, *θ* decreases, 15 < Li^+^ concentration ≤20 mol%) [Fig. 11(c)] due to its small ionic radius, leading to the contraction or expansion of unit cell. Both the contraction and expansion of unit cell would reduce the symmetry of crystal field around Ln ions, inducing the sharp increase of pure red UC luminescence intensity^29^,^30^,^31^. The strongest UC luminescence intensity is acquired in β-NaLuF_4_:20% Yb^3+^, 1% Tm^3+^, 20% Er^3+^ with 15 mol% Li^+^ doping, which is attributed to the most asymmetric surrounding environment around Ln ions, as shown in Fig. 9(b).

## Conclusion

In summary, hexagonal phase microdisks, microprisms, and microtubes were achieved by simply changing the amount of citric acid in the initial reaction solution. Pure red UC luminescence can be observed in β-NaLuF_4_:Yb^3+^, Tm^3+^, Er^3+^ and Li^+^ doped β-NaLuF_4_:20% Yb^3+^, 1% Tm^3+^, 20% Er^3+^. We prove that the low-content doping of Tm^3+^ and high-content doping of Er^3+^ induce great enhancement in the RGR. The RGR in 0.5% Tm^3+^ (R/G = 43.7) and 1% Tm^3+^ (R/G = 49.3)-groups with 20% Er^3+^ doping are increased by 26 and 19 times compared to their 0.5% Er^3+^ doping (R/G = 1.66, 2.57). Green emission varies from one-photon process to two-photon processes, and red emission (660 nm) keeps one-photon process in 0.5% Tm^3+^ and 1% Tm^3+^-groups. Based on the rate equations, we report the theoretical model about the pure red UC mechanism in Yb^3+^/Tm^3+^/Er^3+^ doped system. It is proposed that the pure red UC luminescence is mainly ascribed to the ETU from Tm^3+^:^3^*F*_4_ → ^3^*H*_6_ to Er^3+^:^4^*I*_11/2_ → ^4^*F*_9/2_ and the CR effect [Er^3+^:^4^*S*_3/2_ + ^4^*I*_15/2_ → ^4^*I*_9/2_ + ^4^*I*_13/2_ (CR3)] rather than the long-accepted and most popular mechanism (CR1 process among Er^3+^:^4^*F*_7/2_ + ^4^*I*_11/2_ → ^4^*F*_9/2_ + ^4^*F*_9/2_). Additionally, compared to the Li^+^-free sample, the pure red UC luminescence in β-NaLuF_4_:20% Yb^3+^, 1% Tm^3+^, 20% Er^3+^ with 15 mol% Li^+^ doping is enhanced by 13.7 times. The results suggest that the enhanced pure red UC luminescence in Li^+^ doped β-NaLuF_4_:20% Yb^3+^, 1% Tm^3+^, 20% Er^3+^ may have potential applications in flat-panel displays, solid-state lasers and light-emitting diodes. Besides, this study provides a general and effective approach to obtain intense pure red UC luminescence, which can be applied to other synthetic strategies to prepare many types of nanocrystals with high purity of red UC luminescence, making it suitable for the future bioapplications.

## Methods

### Chemicals

All of the chemicals are of analytical grade and used as received without further purification. 1 M of Lu(NO_3_)_3_, 0.5 M of Yb(NO_3_)_3_, 0.1 M of Er(NO_3_)_3_, and 0.1 M of Tm(NO_3_)_3_ stock solutions were prepared by dissolving the corresponding rare earth oxide (99.99%) in dilute nitric acid (30%) at elevated temperature.

### Preparation

A series of β-NaLuF_4_ crystals with different morphologies were synthesized via a hydrothermal method using citric acid as a chelating agent. In a typical procedure, (2 mmol/3 mmol/8 mmol) of citric acid (2 M, 1 mL/1.5 mL/4 mL), 5 mmol of NaOH (4 M, 1.25 mL) and 10 mL of deionized water were mixed and stirred for 10 min. Then 1 mmol of Ln(NO_3_)_3_ [1 mmol of Lu(NO_3_)_3_ (1 M, 1 mL)] was added to the above mixture and then stirred for 30 min to form the RE-Cit^3−^ complex. Subsequently, an aqueous solution containing 8 mmol of NaF (1 M, 8 mL) and (9 mL/8.5 mL/6 mL) of deionized water was added into the chelated RE-Cit^3-^ complex to form a colloidal suspension and kept stirring for another 30 min. Finally, the suspension was transferred into a 50 mL-Teflon vessel, sealed in an autoclave and maintained at 200 °C for 10 h before cooling down naturally. The final products were separated by centrifugation, washed several times with ethanol and deionized water, then dried in air at 60 °C for 12 h. β-NaLuF_4_: 20% Yb^3+^, (0%/0.5%/1%)Tm^3+^, (0.5%/2%/5%/10%/20%)Er^3+^ crystals and (8%/15%/20%)Li^+^ doped β-NaLuF_4_:20% Yb^3+^, 1% Tm^3+^, 20% Er^3+^ crystals were prepared by a similar process (Cit^3−^ = 3 mmol) under the same experimental conditions. In particular, as for (8%/15%/20%)Li^+^ doped β-NaLuF_4_:20% Yb^3+^, 1% Tm^3+^, 20% Er^3+^ crystals, after the formation of RE-Cit^3−^ complex, a mixture containing (0.64 mmol/1.2 mmol/1.6 mmol) of LiNO_3_ (1 M, 0.64 mL/1.2 mL/1.6 mL), (7.36 mmol/6.8 mmol/6.4 mmol) of NaF (1 M, 7.36 mL/6.8 mL/6.4 mL), (0.32 mmol/0.6 mmol/0.8 mmol) of NH_4_HF_2_ (1 M, 0.32 mL/0.6 mL/0.8 mL) and (6 mL/5.5 mL/5.5 mL) of deionized water were added into the chelated RE-Cit^3−^ complex to form the colloidal suspension.

### Characterization

The phase and structure of the as-prepared products were confirmed by powder X-ray diffraction (XRD) patterns using the D-Max 2200VPC XRD from Rigaku Company. Morphologies and grain sizes were verified by using an Oxford Quanta 400F Thermal Field Emission environmental Scanning Electronic Microscope (SEM). UC photoluminescence spectra were acquired on the Edinburgh Instrument FLSP920 steady-state fluorescence spectrometer equipped with a 2 W 980 nm laser diode. The spot size of the 980 nm laser on the samples is about 0.05 cm^2^.

## Additional Information

**How to cite this article**: Lin, H. *et al*. Morphology evolution and pure red upconversion mechanism of β-NaLuF_4_ crystals. *Sci. Rep.* **6**, 28051; doi: 10.1038/srep28051 (2016).

## Figures and Tables

**Table 1:** Summary of RGR in (a) β-NaLuF_4_:20% Yb/*x*Er, (b) β-NaLuF_4_:20% Yb/0.5% Tm/*x*Er, and (c) β-NaLuF_4_:20% Yb/1% Tm/*x*Er (*x* = 0.5, 2, 5, 10, 20%).

(a)	R/G	(b)	R/G	(c)	R/G
20% Yb/0.5% Er	**5.93**	20% Yb/0.5% Tm/0.5% Er	**1.66**	20% Yb/1% Tm/0.5% Er	**2.57**
20% Yb/2% Er	**4.16**	20% Yb/0.5% Tm/2% Er	**3.46**	20% Yb/1% Tm/2% Er	**2.93**
20% Yb/5% Er	**3.66**	20% Yb/0.5% Tm/5% Er	**9.70**	20% Yb/1% Tm/5% Er	**10.6**
20% Yb/10% Er	**3.20**	20% Yb/0.5% Tm/10% Er	**25.6**	20% Yb/1% Tm/10% Er	**31.5**
20% Yb/20% Er	**4.50**	20% Yb/0.5% Tm/20% Er	**43.7**	20% Yb/1% Tm/20% Er	**49.3**
